# Association between use of liraglutide and liver fibrosis in patients with type 2 diabetes

**DOI:** 10.3389/fendo.2022.935180

**Published:** 2022-08-10

**Authors:** Yijiong Tan, Qin Zhen, Xiaoying Ding, Tingting Shen, Fang Liu, Yufan Wang, Qidi Zhang, Renkun Lin, Lili Chen, Yongde Peng, Nengguang Fan

**Affiliations:** ^1^ Department of Clinical Pharmacy, Shanghai General Hospital, Shanghai Jiao Tong University, Shanghai, China; ^2^ Department of Endocrinology and Metabolism, Shanghai General Hospital, Shanghai Jiao Tong University, Shanghai, China; ^3^ Department of Gastroenterology, Shanghai General Hospital, Shanghai Jiao Tong University, Shanghai, China

**Keywords:** nonalcoholic fatty liver disease, type 2 diabetes mellitus, liver fibrosis, obesity, liraglutide

## Abstract

**Objective:**

Patients with type 2 diabetes have a high risk of non-alcoholic fatty liver disease (NAFLD) and related liver fibrosis. Glucagon-like peptide-1 receptor agonists (GLP-1RAs) have demonstrated efficacy in improving NAFLD, while their effectiveness on liver fibrosis is limited in type 2 diabetic patients.

**Materials/Methods:**

A prospective cohort study was performed in type 2 diabetic patients. The study subjects were divided into two groups based on the use of liraglutide or not, and propensity score matching (PSM) was also conducted. After 12 months follow-up, liver fibrosis was assessed by NAFLD fibrosis score (NFS) fibrosis-4 (FIB-4), and liver stiffness measurement (LSM). The association between liraglutide use and liver fibrosis was analyzed by multivariable linear regression.

**Results:**

In the current study, a total of 1,765 type 2 diabetic patients were enrolled. 262 patients were liraglutide user and 1,503 were nouser. After 12 months follow-up, liraglutide use tended to be associated with reduced prevalence of advanced fibrosis (3.1% vs. 6.1%, *P* = 0.218). After adjustment for confounding factors, multivariable linear regression revealed that liraglutide use was negatively associated with decreased NFS (β= -0.34, *P* = 0.043), FIB4 (β= -0.26, *P* = 0.044) and LSM (β= -4.95, *P* = 0.007) in type 2 diabetics. The results after PSM were similar to those before PSM.

**Conclusions:**

Liraglutide treatment is associated with decreased liver fibrosis in type 2 diabetic subjects.

## Introduction

Nonalcoholic fatty liver disease (NAFLD) is the most frequent chronic liver disorder worldwide, which includes a range of pathological conditions from simple steatosis to nonalcoholic steatohepatitis (NASH), fibrosis and even hepatocellular carcinoma (HCC) (1). The incidence of NAFLD is rising along with obesity and type 2 diabetes mellitus (T2DM). NAFLD is estimated to affect up to 25% of the general population and 70%–80% of people with T2DM (1–3). T2DM further promotes the progression of NAFLD from simple steatosis to NASH and fibrosis (3).

During the last decade, it has grown increasingly evident that hepatic fibrosis is the strongest predictor of NAFLD-related morbidity and mortality (4, 5). The presence of clinically relevant liver fibrosis (F2 to F4) can occur in up to 15% of those with NAFLD and T2DM (6). Early recognition and treatment of NAFLD and liver fibrosis in people with T2DM are crucial. Although liver biopsy remains the gold-standard diagnosis of fibrosis, several non-invasive indices including NAFLD fibrosis score (NFS) and fibrosis-4 (FIB-4) can be used to estimate the prevalence and extent of fibrosis (7, 8). With regard to the treatment of NAFLD, up to date the pharmacological therapy of NAFLD and related liver fibrosis is still rare.

Glucagon-like peptide-1 receptor agonists (GLP-1RAs) are subcutaneous antidiabetic drugs approved for the treatment of T2DM. They are also effective in reducing both body weight and visceral adipose tissue, and have beneficial effects on the risk of cardiovascular and renal outcomes (9–13). In addition, recent evidence has shown that GLP-1RAs also improve hepatic histological components of NAFLD (14–16). Liraglutide and semaglutide consistently resolved NASH histologically in 40% to 60% of patients (17, 18). However, their effects on fibrosis in NAFLD were inconsistent (17, 18). Thus, it remains to be determined whether GLP-1RAs have ameliorative effects on NAFLD related liver fibrosis.

The present study was therefore conceived to explore the association between liraglutide use and liver fibrosis related to NAFLD in an unselected sample of adults with T2DM.

## Methods

### Subjects

All subjects were enrolled from the department of Endocrinology and Metabolism at Shanghai General Hospital from May 2017 to June 2021. Diagnosis of type 2 diabetes was based on the 1999 World Health Organization criteria. A standard questionnaire was distributed to all participants, which asked questions about present and past illnesses and medical treatment, and subjects with an alcohol intake >140 grams per week for men and 70 grams per week for women, with hepatitis, auto-immune hepatitis, or any other chronic liver disease, and with the treatment of pioglitazone and other GLP-1RAs rather than liraglutide were excluded from the study. The subjects were followed for 12 months and data was collected at baseline and 12 months later. In the end, 1,765 type 2 diabetic patients were included in the final analysis. The Institutional Review Board of Shanghai General Hospital affiliated to Shanghai Jiao Tong University School of Medicine approved this study, which was performed in accordance with the principle of the Helsinki Declaration II. Written informed consent was obtained from all subjects.

### Anthropometric and biochemical measurements

Body weight, height, systolic and diastolic blood pressure (SBP, DBP) were measured after overnight fasting for at least 8 hours. BMI was calculated by dividing the body weight by the square of height in meters.

A nurse with extensive experience collected blood samples. Biochemical parameters including serum triglycerides (TG), total cholesterol (TC), low-density lipoprotein cholesterol (LDL-C), high-density lipoprotein cholesterol (HDL-C), alanine aminotransferase (ALT), aspartate aminotransferase (AST), serum creatinine (Scr) and serum uric acid (SUA) were measured using an autoanalyzer (Beckman, Palo Alto, CA). Blood glucose were measured with glucose oxidase method and HbA1c was evaluated by high-performance liquid chromatography.

### Non-invasive assessments of liver fibrosis

NFS was calculated according previous study: −1.675 + 0.037 × age (years) + 0.094 × BMI (kg/m^2^) + 1.13 × IFG/diabetes (yes = 1, no = 0) + 0.99 × AST/ALT ratio − 0.013 × platelet (×10^9^/L) − 0.66 × albumin (g/dL) (7). As all subjects in the present study were diabetic, so NFS = −1.675 + 0.037 × age (years) + 0.094 × BMI (kg/m^2^) + 1.13 + 0.99 × AST/ALT ratio − 0.013 × platelet (×10^9^/L) − 0.66 × albumin (g/dL). FIB-4 was calculated as follow:  (age (years) × AST (U/L))/(platelet count (× 10^9^/L) × ALT (U/L)1/2) (8). In addition, liver stiffness measurement (LSM) was performed using Fibroscan (Echosens^®^, Paris, France).

### Statistical analysis

All statistical analyses were performed using SPSS 13.0 (Chicago, IL). Continuous variables were presented as means ± SD or median (interquartile range). Differences among groups were tested by *t* test for continuous variables and *x* ^2^ test for categorical variables. Multivariate linear regression model was performed to evaluate the independent association between liraglutide use and liver fibrosis assessed by NFS. *P* < 0.05 was considered statistically significant.

## Results

### Baseline clinical characteristics of the subjects before and after matching

Among the 1,765 type 2 diabetic patients, 262 were taking liraglutide users and 1,503 were nousers. Clinical characteristics of the subjects according to the use of liraglutide were summarized in **Table 1**. Proportion of female, BMI, DBP, duration of diabetes, ALT, AST, UA, HbA1C was significantly higher, while age, HDL-C was lower in the user of liraglutide when compared with the nousers (all *P* < 0.05). There was no significant difference in SBP, FBG, Scr, TC, LDL-C and NFS between the two groups.

**Table 1:** Baseline data in the population according the use of liraglutide before PSM.

Characteristics	Liraglutide nonusers	Liraglutide users	*P* value
Number	1503	262	
Age (years)	50.7 ± 11.9	47.8 ± 12.9	<0.001
DBP (mmHg)	77.1 ± 10.8	79.0 ± 10.1	0.007
SBP (mmHg)	129.3 ± 17.3	128.8 ± 16.5	0.677
BMI (kg/m^2^)	25.2 ± 3.3	29.3 ± 3.4	<0.001
Duration (months)	53.1 ± 73.0	80.4 ± 85.2	<0.001
FBG (mmol/L)	8.3 ± 3.1	8.1 ± 2.8	0.351
HbA1c (%)	8.5 ± 2.2	8.8 ± 2.0	0.045
ALT (IU/L)	30.2 ± 30.8	36.6 ± 28.6	0.002
AST (IU/L)	23.9 ± 19.1	27.4 ± 19.9	0.007
Scr (μmol/L)	68.6 ± 205.0	64.1 ± 27.6	0.727
UA (μmol/L)	339.5 ± 100.7	361.2 ± 99.7	0.001
TG (mmol/L)	2.2 ± 3.2	2.6 ± 2.7	0.05
TC (mmol/L)	4.8 ± 1.4	4.8 ± 1.5	0.555
HDL-C (mmol/L)	1.0 ± 0.3	0.9 ± 0.2	<0.001
LDL-C (mmol/L)	2.8 ± 1.0	2.7 ± 1.0	0.14
NFS	-1.2 ± 1.2	-1.2 ± 1.3	0.689
Sex (n,%)			0.002
Female	471 (31.3%)	108 (41.2%)	
Male	1032 (68.7%)	154 (58.8%)	
Current smoking (n,%)			0.028
0	787 (69.8%)	166 (77.2%)	
1	341 (30.2%)	49 (22.8%)	
Current drinking (n,%)			0.787
0	701 (51.7%)	128 (52.7%)	
1	654 (48.3%)	115 (47.3%)	
Metformin (n,%)			<0.001
0	506 (33.7%)	41 (15.6%)	
1	997 (66.3%)	221 (84.4%)	
Akabose (n,%)			<0.001
0	1011 (67.3%)	119 (45.4%)	
1	492 (32.7%)	143 (54.6%)	
DPP-4i (n,%)			<0.001
0	537 (35.7%)	262 (100.0%)	
1	966 (64.3%)	0 (0.0%)	
SGLT-2i (n,%)			<0.001
0	1282 (85.3%)	165 (63.0%)	
1	221 (14.7%)	97 (37.0%)	
Sulfonylurea (n,%)			<0.001
0	1203 (80.0%)	245 (93.5%)	
1	300 (20.0%)	17 (6.5%)	
Insulin (n,%)			<0.001
0	1100 (73.2%)	162 (61.8%)	
1	403 (26.8%)	100 (38.2%)	

DPP-4i, dipeptidyl peptidase-4 inhibitor; GLP1-RAs, glucagon-like peptide 1 receptor agonists; HbA1c, hemoglobin A1c; SGLT-2i, sodium glucose co-transporters 2 inhibitor; TZD, thiazolidinedione.

Furthermore, the subjects were propensity score matching (PSM) (1:1) according to the age, sex and BMI of the subjects. Clinical characteristics of the matched population was exhibited in **Table 2**, liraglutide users and nousers had similar age and sex proportion, and the difference of BMI was decreased when compared with the difference before PSM.

**Table 2:** Baseline data in the population according the use of GLP-1RAs after PSM.

Characteristics	Liraglutide nonusers	Liraglutide users	*P* value
Number	254	254	
Age (years)	49.0 ± 13.2	47.8 ± 12.9	0.313
BMI (kg/m^2^)	27.8 ± 4.3	29.1 ± 3.3	<0.001
DBP (mmHg)	78.8 ± 11.5	79.1 ± 10.0	0.783
SBP (mmHg)	133.0 ± 17.1	128.8 ± 16.6	0.006
Duration (months)	40.8 ± 58.5	81.0 ± 85.9	<0.001
FBG (mmol/L)	8.6 ± 3.2	8.2 ± 2.8	0.107
HbA1c (%)	8.6 ± 2.2	8.8 ± 2.0	0.314
ALT (IU/L)	37.0 ± 58.0	36.9 ± 28.9	0.978
AST (IU/L)	28.6 ± 38.6	27.4 ± 20.0	0.654
Scr (μmol/L)	61.2 ± 15.5	64.1 ± 27.6	0.148
UA (μmol/L)	350.5 ± 94.6	361.3 ± 99.3	0.212
TC (mmol/L)	4.9 ± 1.4	4.8 ± 1.5	0.311
TG (mmol/L)	2.2 ± 1.8	2.6 ± 2.7	0.071
HDL-C (mmol/L)	1.0 ± 0.2	0.9 ± 0.2	<0.001
LDL-C (mmol/L)	2.9 ± 0.9	2.7 ± 1.0	0.037
NFS	-1.1 ± 1.3	-1.2 ± 1.3	0.523
FIB-4	1.2 ± 0.8	1.1 ± 0.7	0.042
Sex (n,%)			0.716
0	98 (38.6%)	102 (40.2%)	
1	156 (61.4%)	152 (59.8%)	
Current smoking (n,%)			0.614
0	184 (72.7%)	189 (74.7%)	
1	69 (27.3%)	64 (25.3%)	
Current drinking (n,%)			0.428
0	173 (68.9%)	166 (65.6%)	
1	78 (31.1%)	87 (34.4%)	
Metformin (n,%)			<0.001
0	72 (28.3%)	40 (15.7%)	
1	182 (71.7%)	214 (84.3%)	
Akarbose (n,%)			<0.001
0	202 (79.5%)	115 (45.3%)	
1	52 (20.5%)	139 (54.7%)	
DPP-4i (n,%)			<0.001
0	106 (41.7%)	254 (100.0%)	
1	148 (58.3%)	0 (0.0%)	
SGLT-2i (n,%)			<0.001
0	209 (82.3%)	161 (63.4%)	
1	45 (17.7%)	93 (36.6%)	
Sulfonylurea (n,%)			<0.001
0	210 (82.7%)	237 (93.3%)	
1	44 (17.3%)	19 (6.7%)	
Insulin (n,%)			<0.001
0	202 (79.5%)	156 (61.4%)	
1	52 (20.5%)	98 (38.6%)	

DPP-4i, dipeptidyl peptidase-4 inhibitor; GLP1-RAs, glucagon-like peptide 1 receptor agonists; HbA1c, hemoglobin A1c; SGLT-2i, sodium glucose co-transporters 2 inhibitor; TZD, thiazolidinedione.

### Effects of liraglutide on clinical data and the prevalence of advanced liver fibrosis

**Figure 1:**
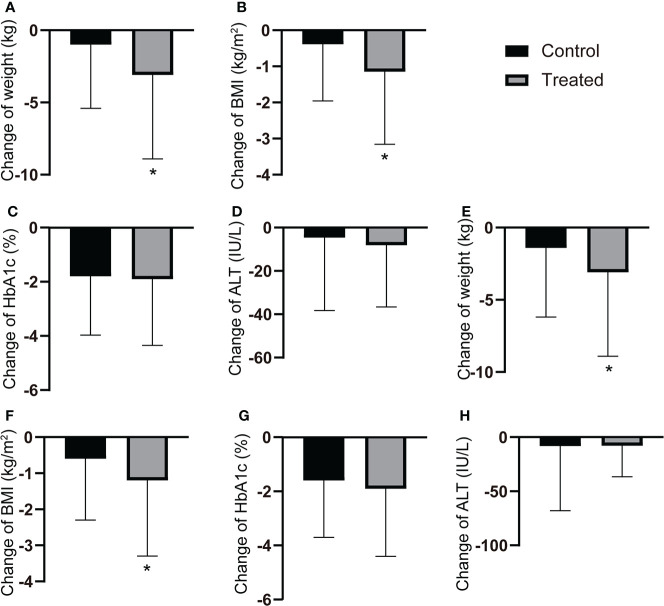
Changes of the clinical characteristics after 12-months follow-up. **(A-D)** The changes of body weight, BMI, HbA1c and ALT after 12-month follow-up in all T2DM patients with or without GLP-1RAs use. **(E-H)** The changes of body weight, BMI, HbA1c and ALT after 12-month flow-up in T2DM patients with or without GLP-1RAs use after PSM. *P < 0.05.

After a 12-month follow-up, liraglutide users showed a significant reduction in body weight and BMI compared to the control group (all *P* < 0.05). In contrast, there was no significant differences in HbA1c and ALT (**Figures 1A–D**). After PSM, similar results were observed in the cohort (**Figures 1E–H**).

**Figure 2:**
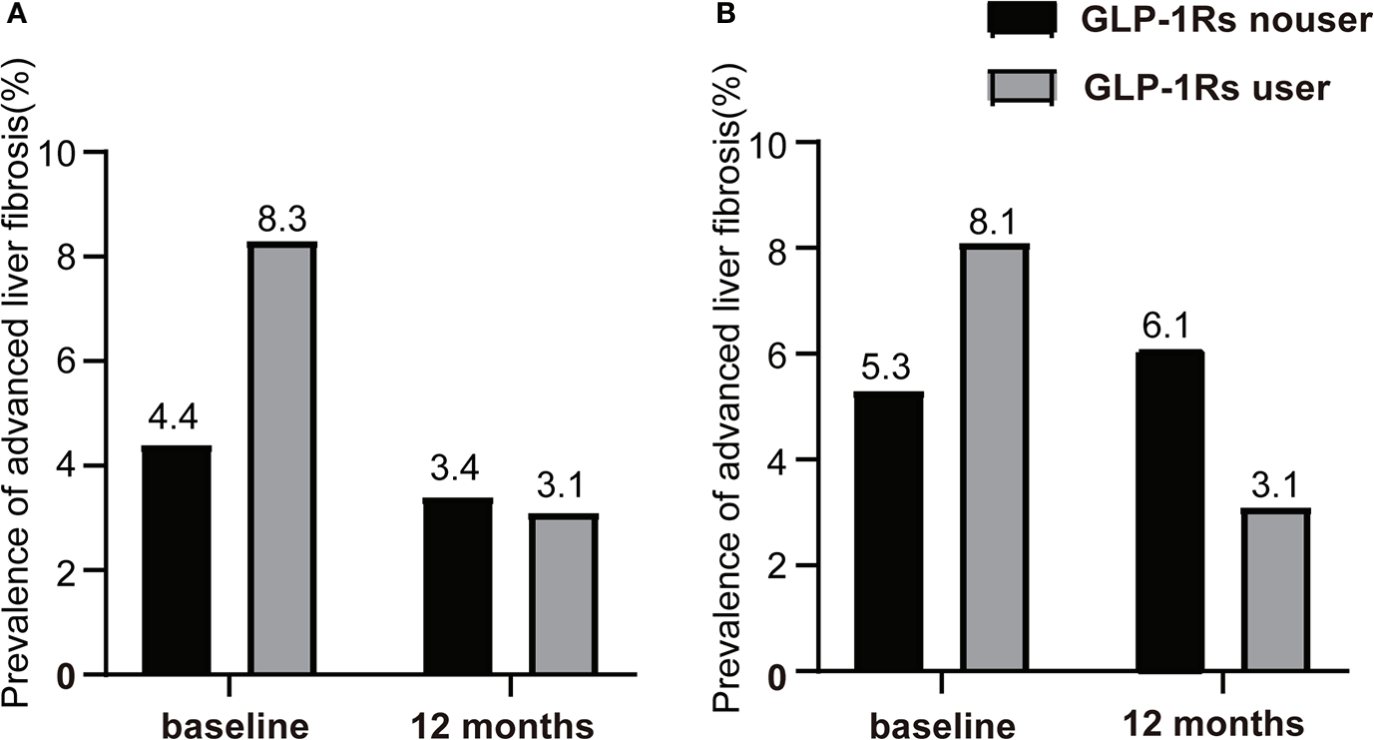
Change of the prevalence of advanced liver fibrosis after 12-month follow-up. **(A)** The prevalence of advanced liver fibrosis at baseline and 12-month follow-up in all T2DM patients with or without GLP-1RAs use. **(B)** The prevalence of advanced liver fibrosis at baseline and 12-month follow-up in T2DM patients with or without GLP-1RAs use after PSM.

Next, liver fibrosis was evaluated by NFS and the overall prevalence of advanced liver fibrosis (NFS > 0.676) was 5.0%. At baseline, the prevalence of advanced liver fibrosis in the control and liraglutide group was 4.4% and 8.3%, respectively (P < 0.01). After 12 months treatment, the prevalence of advanced liver fibrosis in the two group was comparable (3.4% vs. 3.1%, *P* > 0.05) (**Figure 2A**). In the cohort after PSM, the prevalence of advanced liver fibrosis in the control and liraglutide group at baseline was 5.3% and 8.1%, respectively (*P* > 0.05). After 1 year treatment, the prevalence of advanced liver fibrosis was 6.1%, while that in the liraglutide group was decreased to 3.1%, though the difference was not significant probably due to the limit of sample size (*P* = 0.218) (**Figure 2B**).

### Association of liraglutide use with liver fibrosis

Using multivariate linear regression, we further studied the independent association between liraglutide use and noninvasive liver fibrosis markers including NFS and FIB-4. As shown in **Table 3**, liraglutide use was negatively associated with NFS after adjustment for age, sex, BMI, SBP, DBP, smoking, drinking and duration of diabetes (model 1). After further adjustment for FBG, HbA1c, TG, TC, LDL-C and HDL-C (model 2), liraglutide use remained significantly correlated with NFS. Finally, additional adjustment of the use of other antidiabetic medicines including metformin, SGLT2i, sulfonylurea, DPP-4i and insulin also did not significantly change the association between liraglutide use and NFS (model 3). The association between liraglutide and NFS was further analyzed after PSM, and the results were consistent with those before PSM (**Table 3**). Consistently, use of liraglutide was also negatively associated with FIB-4 before and after PSM (**Table 4**).

**Table 3:** Association between GLP-1RAs use and NFS before and PSM.

Before PSM	β	95%CI	*P*
Model 1	-0.2	-0.45, 0.04	0.104
Model 2	-0.32	-0.62, -0.02	0.037
Model 3	-0.34	-0.67, -0.01	0.043
**After PSM**			
Model 1	-0.3	-0.54, -0.07	0.012
Model 2	-0.3	-0.55, -0.05	0.018
Model 3	-0.27	-0.54, -0.01	0.045

**Table 4:** Association between GLP-1RAs use and FIB-4 before and after PSM.

Before PSM	β	95%CI	*P*
Model 1	-0.17	-0.34, -0.01	0.048
Model 2	-0.24	-0.48, -0.01	0.045
Model 3	-0.26	-0.51, -0.01	0.044
**After PSM**			
Model 1	-0.23	-0.42, -0.03	0.022
Model 2	-0.23	-0.43, -0.04	0.021
Model 3	-0.25	-0.46, -0.04	0.021

Furthermore, we investigated the effect of liraglutide on LSM performed by transient elastography. As expected, treatment of liraglutide was associated with reduced LSM after adjustment of confounders (β: -4.95; 95%CI:-8.43, -1.47; *P*=0.007).

## Discussion

NAFLD related liver fibrosis affects a large proportion of individuals with T2DM. Nonetheless, to date, no medicine has been approved for the treatment NAFLD and liver fibrosis. In the present study, we explored the association of liraglutide use and liver fibrosis in T2DM patients. It was found that liraglutide use was negatively associated with liver fibrosis in patients with T2DM.

NAFLD and related liver fibrosis is common in patients with T2DM. In one recent study, the prevalence of NAFLD in T2DM patients was 70%, while advanced liver fibrosis is 9% (6). In another meta-analysis, the prevalence of NAFLD in patients with T2DM was 55.5%, while the prevalence of advanced fibrosis is 17.0%. In our study, NFS was used as a marker of liver fibrosis and the prevalence of advanced liver fibrosis in T2DM was 5.0%. Different populations and methods to assess liver fibrosis may be responsible for the inconsistency in the above studies. In fact, T2DM has been recognized a promoter of liver fibrosis. Though the underlying mechanisms remain largely unknown, insulin resistance and hyperglycemia may contribute to liver fibrosis in T2DM patients (19, 20).

GLP-1RAs are widely used in the treatment of T2DM and are effective in lowering blood glucose level and body weight. In recent years, the role of GLP-1RAs in NAFLD has also been investigated. The role of GLP-1RAs in hepatic steatosis and NASH is demonstrated in the previous studies (15, 16, 18). In contrast, its role in liver fibrosis in NAFLD is still controversial. In the Liraglutide Efficacy and Action in Non-alcoholic steatohepatitis (LEAN) randomized phase 2 trial, liraglutide improved NASH as well as reduced fibrosis progression both in diabetics and non-diabetics (15). More recently, a phase 2 study of semaglutide, a longer-acting GLP-1RA has also shown to effectively reduce liver enzymes and amealioate NASH after 72 weeks of therapy. Nevertheless, the study failed to show any significant improvement in fibrosis stage (18). In another real-world study, GLP-1RAs use was shown to improve markers of liver fibrosis in T2DM (21). Consistently, we observed decreased prevalence of advance liver fibrosis in T2DM individuals treated with liraglutide compared with nousers. In addition, liraglutide use was negatively associated with NFS, FIB-4 and LSM, three noninvasive assessments of liver fibrosis. Thus, our study provides additional clinical evidence of a possible role of GLP-1RAs in the treatment of liver fibrosis in T2DM patients.

The mechanism of GLP-1RAs’ role on NAFLD is still not well illustrated. Reduction in body weight is one of the reason responsible for the favorable effect of GLP-1RAs on NAFLD. Besides, GLP-1RAs have anti-inflammatory and antioxidant properties and contributed to significant reductions in biomarkers of inflammation and oxidative stress in clinical trials (22). In animal models, GLP-1RAs treatment could alleviate inflammation in liver (especially M1 pro-inflammatory macrophages accumulation) (23). These properties of GLP-1RAs could confer its protection against liver fibrosis.

The present study has several limitations that need to be considered. First, the follow up period was relative short, which may affect the association between liraglutide use and liver fibrosis. Second, this study did not include a diagnosis of NAFLD, which precluded stratifying by the presence or absence of NAFLD. Third, fibrosis was not evaluated by liver biopsy. Nevertheless, NFS, FIB-4 and LSM has become widely used detect liver fibrosis.

## Conclusion

In conclusion, the present study showed that GLP-1RA use was negatively correlated with liver fibrosis in type 2 diabetic patients. GLP-1RAs may be a therapy to ameliorate liver fibrosis in type 2 diabetic patients.

## Data availability statement

The raw data supporting the conclusions of this article will be made available by the authors, without undue reservation.

## Ethics statement

The studies involving human participants were reviewed and approved by The Institutional Review Board of Shanghai General Hospital affiliated to Shanghai Jiao Tong University School of Medicine. The patients/participants provided their written informed consent to participate in this study.

## Author contributions

Study design: NF and YP; Collection and assembly of data: YT and QZ; Data analysis and interpretation: All authors; Manuscript writing: All authors; Final approval of manuscript: All authors.

## Funding

This study was supported by the Natural Science Foundation of China (81870596, 81400785) , the Shanghai Natural Science Foundation (21ZR1451200), Clinical research plan of SHDC [No.SHDC2020CR1016B], Shanghai Jiao Tong University Research Funding on Medical, Engineering Interdisciplinary Project (YG2019GD05), Multi-center Clinical Research Project of Shanghai Jiao Tong University School of Medicine (DLY201824), The third round cooperation project of Songjiang district municipal Health Commission (0702N18003), Shanghai General Hospital Clinical Research Innovation Team Project (CTCCR-2018A02).

## Conflict of interest

The authors declare that the research was conducted in the absence of any commercial or financial relationships that could be construed as a potential conflict of interest.

## Publisher’s note

All claims expressed in this article are solely those of the authors and do not necessarily represent those of their affiliated organizations, or those of the publisher, the editors and the reviewers. Any product that may be evaluated in this article, or claim that may be made by its manufacturer, is not guaranteed or endorsed by the publisher.
